# Homeostatic model assessment of adiponectin (HOMA-Adiponectin) as a surrogate measure of insulin resistance in adolescents: Comparison with the hyperglycaemic clamp and homeostatic model assessment of insulin resistance

**DOI:** 10.1371/journal.pone.0214081

**Published:** 2019-03-25

**Authors:** Cleliani de Cassia da Silva, Mariana Porto Zambon, Ana Carolina Junqueira Vasques, Daniella Fernandes Camilo, Ana Maria De Bernardi Rodrigues, Maria Ângela Reis de Góes Monteiro Antonio, Ana Raimunda Dâmaso, Sergio Tufik, Marco Tulio de Mello, Raquel Munhoz da Silveira Campos, Bruno Geloneze, for the Brazilian Metabolic Syndrome Study (BRAMS) Investigators

**Affiliations:** 1 Laboratory of Investigation on Metabolism and Diabetes (Limed), Gastroenterological Diagnosis and Research Center (Gastrocentro), University of Campinas - Unicamp, Campinas, São Paulo, Brazil; 2 Postgraduate Program in Child and Adolescent Health, Faculty of Medical Sciences, University of Campinas - Unicamp, Campinas, São Paulo, Brazil; 3 Department of Pediatrics, University of Campinas - Unicamp, Campinas, São Paulo, Brazil; 4 School of Applied Sciences, University of Campinas - Unicamp, Limeira, São Paulo, Brazil; 5 School of Health and Life Sciences, University Center Our Lady of Patronage, Itu, São Paulo, Brazil; 6 Postgraduate Program of Nutrition, Federal University of São Paulo - Unifesp, São Paulo, São Paulo, Brazil; 7 Department of Psychobiology, Federal University of São Paulo - Unifesp, São Paulo, São Paulo, Brazil; 8 School of Physical Education, Physiotherapy and Occupational Therapy, Federal University of Minas Gerais, Belo Horizonte, Minas Gerais, Brazil; 9 Department of Physiotherapy, Therapeutic Resources Laboratory, Federal University of São Carlos - UFSCar, São Carlos, São Paulo, Brazil; Helmholtz Zentrum München, GERMANY

## Abstract

**Background:**

Studies on adults have reported inverse association between the homeostatic model assessment (HOMA) of adiponectin (HOMA-Adiponectin) and the insulin resistance assessed by the glucose clamp technique. To our knowledge, in the pediatric population this association has not been previously investigated.

**Objectives:**

To evaluate the association between the HOMA-Adiponectin and the insulin resistance assessed by the glucose clamp technique in adolescents, and to compare the accuracy of HOMA-Adiponectin and HOMA-insulin resistance (HOMA-IR) for identifying insulin resistance.

**Methods:**

This was a cross-sectional study of 56 adolescents (aged 10–18 years). Insulin resistance was assessed using the HOMA-IR, HOMA-Adiponectin and the hyperglycaemic clamp technique. The clamp-derived insulin sensitivity index, HOMA-Adiponectin, and HOMA-IR were log-transformed to get closer to a normal distribution before analysis.

**Results:**

In the multivariable linear regression analysis controlling for sex and Tanner stage, HOMA-Adiponectin was inversely associated with the clamp-derived insulin sensitivity index (unstandardized coefficient [B] = -0.441; *P* < 0.001). After additional adjustment for waist circumference-to-height ratio, this association remained significant (B = -0.349; *P* = < 0.001). Similar results were observed when HOMA-IR replaced HOMA-Adiponectin in the model (B = -1.049 and B = -0.968 after additional adjustment for waist circumference-to-height ratio); all *P* < 0.001. The area under the receiver operating characteristic curve for predicting insulin resistance was 0.712 (*P* = 0.02) for HOMA-Adiponectin and 0.859 (*P* < 0.0001) HOMA-IR.

**Conclusions:**

The HOMA-Adiponectin was independently associated with insulin resistance and exhibited a good discriminatory power for predicting it. However, it did not show superiority over HOMA-IR in the diagnostic accuracy.

## Introduction

The fasting homeostatic model assessment of insulin resistance (HOMA-IR) [1] has been used as a surrogate measure of insulin resistance for epidemiological studies in pediatric populations and it assumes that hepatic and peripheral insulin resistance are equal. In 2007, HOMA-Adiponectin was proposed as a surrogate measure of insulin resistance in Japanese adults [2]. Adiponectin levels are reduced in obese and negatively associated with insulin resistance in children and adolescents [3–5]. The inclusion of total serum adiponectin level in the denominator of the HOMA-IR may add an indirect measurement of adiposopathy [6] and, consequently, indirect information of peripheral insulin resistance, giving robustness to its pathophysiological basis.

Studies on adults have reported inverse association between the HOMA-Adiponectin and the insulin resistance assessed by the glucose clamp technique [2, 6]. To our knowledge, in the pediatric population this association has not been previously investigated. Therefore, the purpose of the present study was to evaluate the association between HOMA-Adiponectin and insulin resistance assessed by the glucose clamp technique in adolescents, and to compare the accuracy of HOMA-Adiponectin and HOMA-IR for identifying insulin resistance.

## Methods

### Study population and design

**Fig 1:**
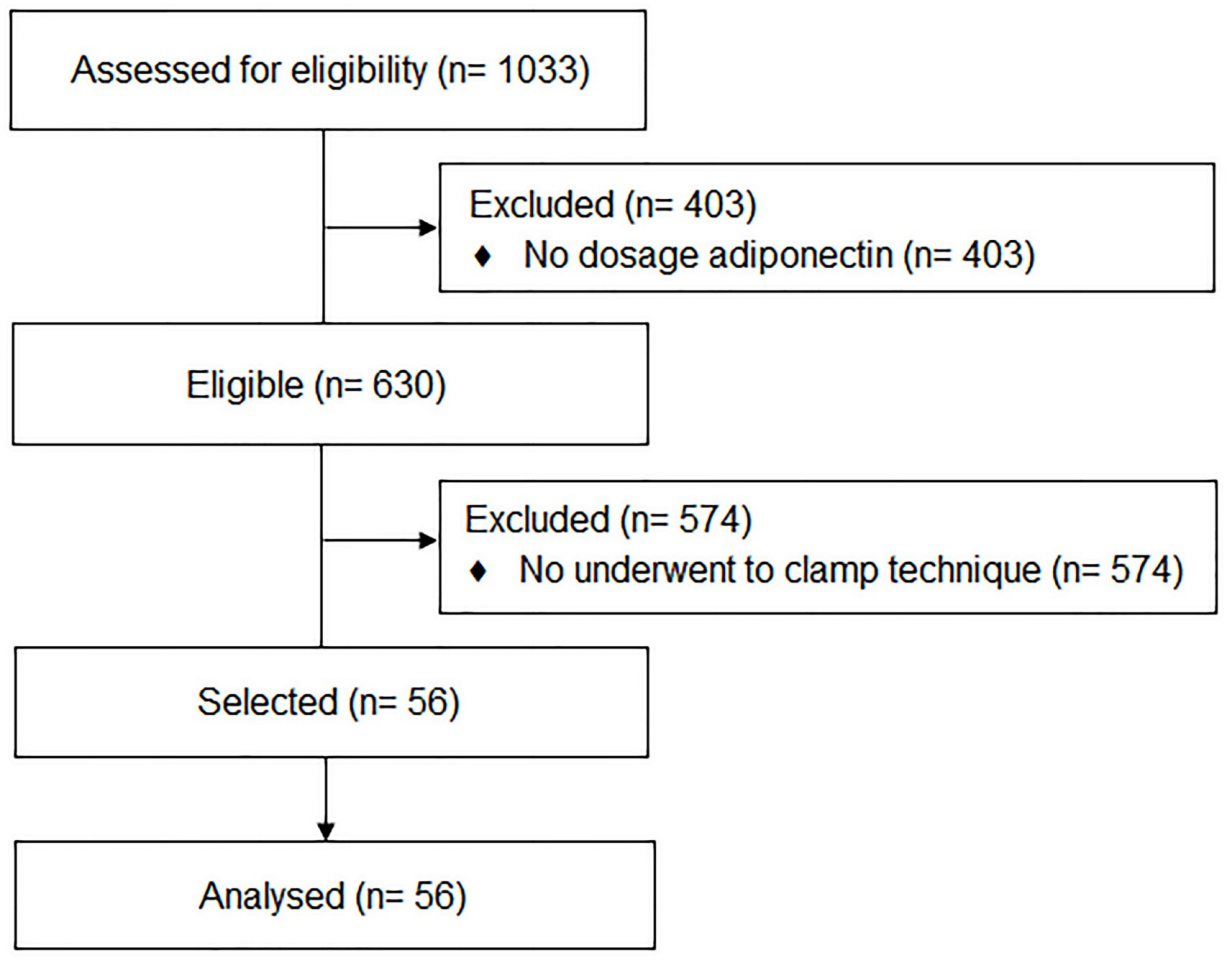
Flow diagram describing the selection of the study population.

This study used data from the Brazilian Metabolic Syndrome Study (BRAMS). All information of the BRAMS Study have previously been described in detail [7, 8]. In brief, BRAMS is a cross-sectional multicenter study that included an intentional non-probabilistic sample of adolescents (10 to 19 years and 11 months) recruited from the Clinic of Obesity in Children and Adolescents at Clinical Hospital of University of Campinas, from the São Paulo Hospital of Federal University of São Paulo, and from the Health Units, public schools and institutions offering educational programs for adolescents. In 2011–2015, about 1033 adolescents were examined. From this cohort, 82 adolescents (10–18 years old) underwent the hyperglycaemic clamp technique. Of the 1033 enrolled participants in the BRAMS study, 630 had valid data for the explanatory variable of interest, HOMA-Adiponectin. Of the 82 adolescents underwent the hyperglycaemic clamp technique, 56 had valid data for the explanatory variable of interest. Fig 1 presents the flow diagram of the study population. Adolescents were eligible to participate if they had a body mass index (BMI) for age and sex at or above the fifth percentile according to the Centers for Disease Control and Prevention growth charts [9]. Adolescents with metabolic disorders (e.g., hypothyroidism, hyperthyroidism, or type 1 and type 2 diabetes), nephropathy, hepatopathy, genetic syndrome, delayed neuropsychomotor development, malnutrition, pregnant or in treatment with systemic corticosteroids and drugs with hypoglycaemic and hypolipidemic properties were not included in the study.

The study was approved by the Ethics Committee of the Faculty of Medical Sciences of University of Campinas (protocol number 900/2010) and conducted in accordance to the Declaration of Helsinki. Written informed consent was obtained from parents.

### Data collection methods

Sexual maturation was self-evaluated [10, 11] according to Tanner criteria [12] and the participants were classified as pubertal (Tanner II-IV) and postpubertal (Tanner V). BMI was calculated as weight in kilograms divided by height in meters squared. BMI-for-age percentile were calculated for each adolescent using Epi Info version 3.5.2 (Centers for Disease Control and Prevention, Atlanta, GA, USA). The nutritional status was classified as normal weight (BMI-for-age between the 5th percentile and the 85th percentile); overweight (BMI-for-age between 85th percentile and the 95th percentile); and obesity (BMI-for-age at or above the 95th percentile) according to the Centers for Disease Control and Prevention criteria (CDC/2000) [9]. Waist circumference was measured directly over the skin, at the midpoint between the lower margin of the last palpable rib and the top of the iliac crest (hip bone), at the end of a normal expiration [13]. Waist circumference-to-height ratio was calculated as the waist circumference (in cm) divided by height (in cm). Lean body mass was estimated by tetrapolar bioimpedance (Biodynamics, model 310, Shoreline, WA, USA). Laboratory assays included plasma glucose (enzymatic colorimetric methods; monoreagent K082, Bioclin Systems II, Quisaba, Bioclin, Belo Horizonte, MG, Brazil), plasma insulin (human insulin enzyme-linked immunosorbent assay kit—EZHI-14K; Millipore, USA; sensitivity: 2 μU/mL; intra-assay coefficients of variation: 4.6–7.0%; inter-assay coefficients of variation: 9.1–11.4%), and the quantitative determination of serum concentrations of total adiponectin (Quantikine Human Adiponectin/Acrp30 ELISA kit; R&D Systems, Inc., Minneapolis, MN; intra-assay coefficients of variation: 2.5–4.7%; inter-assay coefficients of variation: 6.8–6.9%). Insulin resistance was assessed using the HOMA-IR (calculated as the product of the fasting plasma insulin level [in milliunits per liter] and the fasting plasma glucose level [in millimoles per liter], divided by the 22.5) [1], HOMA-Adiponectin (calculated as the product of the fasting plasma insulin level [in milliunits per liter] and the fasting plasma glucose level [in millimoles per liter], divided by the adiponectin level [in microgram per milliliter]) [2], and the 120-min. hyperglycaemic clamp technique [14]. The clamp-derived insulin sensitivity index (ISI) was calculated as the mean exogenous glucose infusion rate from 60–120 minutes of the clamp technique minus urinary glucose excretion, divided by the mean insulin concentration of five determinations during the same time period, and was corrected for lean body mass (LBM) [ISI_LBM_; milligrams of glucose infused per kilogram of lean body mass per minute, multiplied by 100) [14, 15].

### Statistical analysis

The data (S1 File) were analyzed using MedCalc for Windows, version 17.9.7 (MedCalc Software, Ostend, Belgium), and the IBM SPSS Statistics for Windows, version 20.0 (IBM Corporation, Armonk, NY, USA). A *P*-value less than 0.05 was considered statistically significant. The Shapiro-Wilk test was used to evaluate the variables distribution and clamp-derived insulin sensitivity index, HOMA-Adiponectin and HOMA-IR were log-transformed to get closer to a normal distribution. Continuous variables with normal distribution were expressed as mean ± standard deviation, whereas non-normally distributed variables were expressed as median (interquartile ranges). Qualitative data were presented as percentages. Fisher’s exact test was used to compare two proportions. Comparisons of two independent groups were performed using Student’s t-test for groups with normally distributed data, and Mann-Whitney U test for groups with not normally distributed data. Pearson’s correlation coefficient was used to analyze the relationship between two continuous variables. Multiple linear regression analysis was used to examine the associations between clamp-derived insulin sensitivity index (dependent variable) and the HOMA-Adiponectin or HOMA-IR (independent variables). The HOMA-Adiponectin and HOMA-IR were not assessed simultaneously in the regression model because the statistics indicated a multicollinearity problem (variance inflation factor higher than 2 [16], and proportions of variance higher than 0.45 [17]). Like previous studies have shown significant differences in insulin resistance between boys and girls [18]; and that insulin resistance increases significantly at Tanner stage 2, 3, and 4, but decreases to near prepubertal levels at Tanner stage 5 [18]; and that increased abdominal visceral adiposity in obese adolescent is associated with lower insulin sensitivity [19], we included sex, Tanner stage and waist circumference-to-height ratio (a measure of abdominal adiposity) [20, 21], as independent variables in the regression models. Receiver operating characteristic (ROC) analysis was used to evaluate accuracy of the HOMA-Adiponectin for detecting insulin resistance, as evaluated by hyperglycaemic clamp technique, and to compare the diagnostic accuracy of HOMA-Adiponectin versus the HOMA-IR. A comparison between areas under the ROC curve (AUC) was performed, and the method of DeLong et al [22] was used for the calculation of the standard error of the AUCs and of the difference between the two AUCs. Insulin resistance was defined based on cutoff of clamp-derived insulin sensitivity index (lower 10th percentile) derived from the normal-weight group (cutoff, < 0.07 mg/kg_LBM_/min per mU/L x 100). The S2 File contains all analysis codes.

## Results

Overweight/obese adolescents had higher plasma glucose, plasma insulin, HOMA-Adiponectin and HOMA-IR, compared with those with normal weight. Moreover, they had lower adiponectin and clamp-derived insulin sensitivity index compared with those with normal weight (Table 1).

**Table 1:** Participant characteristics.

Variables	Normal weight (n = 22)	Overweight/obese (n = 34)	*P*-value
Age, years	15.6 ± 2.3	14.3 ± 2.2	0.04^a^
Sex, No. (%)			
Female	14 (63.6)	15 (44.1)	0.18^b^
Male	8 (36.4)	19 (55.9)	
Tanner stage, No. (%)			
Pubertal, Tanner II-IV	6 (27.3)	15 (44.1)	0.26^b^
Postpubertal, Tanner V	16 (72.7)	19 (55.9)	
Weight, kg	52.5 (47.6 to 60.2)	82.9 (73.0 to 90.1)	< 0.001^c^
Height, cm	161.0 ± 10.6	165.0 ± 8.5	0.124^a^
Body mass index, kg/m^2^	20.2 (18.8 to 22.8)	28.9 (27.8 to 31.7)	< 0.001^c^
Body mass index-for-age percentile	60.4 (22.5 to 79.9)	97.7 (96.1 to 98.7)	< 0.001^c^
Waist circumference, cm	70.0 (66.3 to 75.5)	95.2 (90.0 to 103.5)	< 0.001^c^
Waist circumference-to-height ratio	0.44 (0.41 to 0.48)	0.57 (0.53 to 0.63)	< 0.001^c^
Lean body mass, kg	39.6 (35.1 to 46.9)	51.7 (48.0 to 61.6)	< 0.001^c^
Adiponectin, μg/mL	5.02 (2.85 to 7.56)	2.44 (1.60 to 4.46)	0.001^c^
Plasma glucose, mg/dL	85 ± 6	89 ± 6	0.03^a^
Plasma insulin, mU/L	7.2 (4.8 to 10.3)	12.0 (8.7 to 18.0)	< 0.001^c^
HOMA-Adiponectin	6.64 (3.49 to 9.15)	22.53 (9.79 to 59.77)	< 0.001^c^
HOMA-IR	1.69 (0.96 to 1.99)	2.63 (1.94 to 3.95)	< 0.001^c^
ISI, mg/kg_LBM_^-1^. min^-1^ per mU/L x 100	0.27 (0.16 to 0.42)	0.09 (0.06 to 0.14)	< 0.001^c^

Abbreviations: HOMA-Adiponectin, homeostasis model assessment of adiponectin; HOMA-IR, homeostasis model assessment of insulin resistance; ISI, insulin sensitivity index; LBM, lean body mass

SI conversion factors: To convert glucose to millimoles per liter, multiply by 0.0555; and to convert insulin to picomoles per liter, multiply by 6

^a^Student’s t-test, data are presented as the mean ± standard deviation;

^b^Fisher’s exact test, data are expressed as a percentage;

^c^Mann-Whitney U test, data are presented as the median (interquartile ranges)

HOMA-Adiponectin and HOMA-IR were inversely correlated with the clamp-derived insulin sensitivity index (r = -0.662; *P* < 0.001 and r = -0.799; *P* < 0.001, respectively).

Multiple linear regression models are presented in Table 2. In the first model that included HOMA-Adiponectin, sex and Tanner stage as the independent variables, HOMA-Adiponectin was inversely associated with the clamp-derived insulin sensitivity index (*P* < 0.001). This model explained 47.1% of the variance in clamp-derived insulin sensitivity. The second model included HOMA-Adiponectin, sex, Tanner stage and waist circumference-to-height ratio as the independent variables. In this model, waist circumference-to-height ratio was inversely associated with the clamp-derived insulin sensitivity index (*P* = 0.026). The inclusion of waist circumference-to-height ratio in the model did not eliminate the inverse significant association between HOMA-Adiponectin and clamp-derived insulin sensitivity index (*P* < 0.001). This model explained 52.1% of the variance in clamp-derived insulin sensitivity. The third model included HOMA-IR, sex and Tanner stage as independent variables. In this model, HOMA-IR was inversely associated with the clamp-derived insulin sensitivity index (*P* < 0.001). This model explained 63.8% of the variance in clamp-derived insulin sensitivity and when we included the waist circumference-to-height ratio (fourth model), it did not increase this variance considerably (64.4%) neither changed the association of HOMA-IR with clamp-derived insulin sensitivity index (*P* < 0.001).

**Table 2:** Multiple regression analysis of the HOMA-Adiponectin and HOMA-IR with clamp-derived insulin sensitivity index.

Dependent variable	Model	Independent variables	B	95% CI	*P*-value	*R*^2^ for model
ISI, mg/kg_LBM_^-1^. min^-1^ per mU/L x 100^a^^,^^b^	1	HOMA-Adiponectin^a^	-0.441	-0.581 to -0.300	< 0.001	0.471
		Sex^c^	0.083	-0.065 to 0.231	0.264	
		Tanner stage^d^	-0.122	-0.276 to 0.032	0.117	
	2	HOMA-Adiponectin^a^	-0.349	-0.506 to -0.192	< 0.001	0.521
		Sex^d^	0.123	-0.23 to 0.269	0.097	
		Tanner stage^d^	-0.125	-0.273 to 0.023	0.096	
		Waist circumference-to-height ratio	-1.018	-1.907 to -0.128	0.026	
	3	HOMA-IR^a^	-1.049	-1.282 to -0.817	< 0.001	0.638
		Sex^d^	-0.011	-0.135 to 0.113	0.856	
		Tanner stage^d^	0.008	-0.125 to 0.142	0.899	
	4	HOMA-IR^a^	-0.968	-1.259 to -0.676	< 0.001	0.644
		Sex^d^	0.011	-0.122 to 0.145	0.867	
		Tanner stage^d^	-0.003	-0.138 to 0.133	0.968	
		Waist circumference-to-height ratio	-0.384	-1.207 to 0.439	0.353	

Abbreviations: B, unstandardized coefficient; CI, confidence interval; ISI, insulin sensitivity index; LBM, lean body mass; HOMA-Adiponectin, homeostasis model assessment of adiponectin; HOMA-IR, homeostasis model assessment of insulin resistance; NA, not applicable

^a^Logarithmic transformation was performed.

^b^Insulin sensitivity index (ISI) expressed as milligrams of glucose infused per kilogram of lean body mass (LBM) per minute.

^c^0 represents the female and 1 the male.

^d^0 represents the postpubertal stage and 1 the pubertal stage

**Fig 2:**
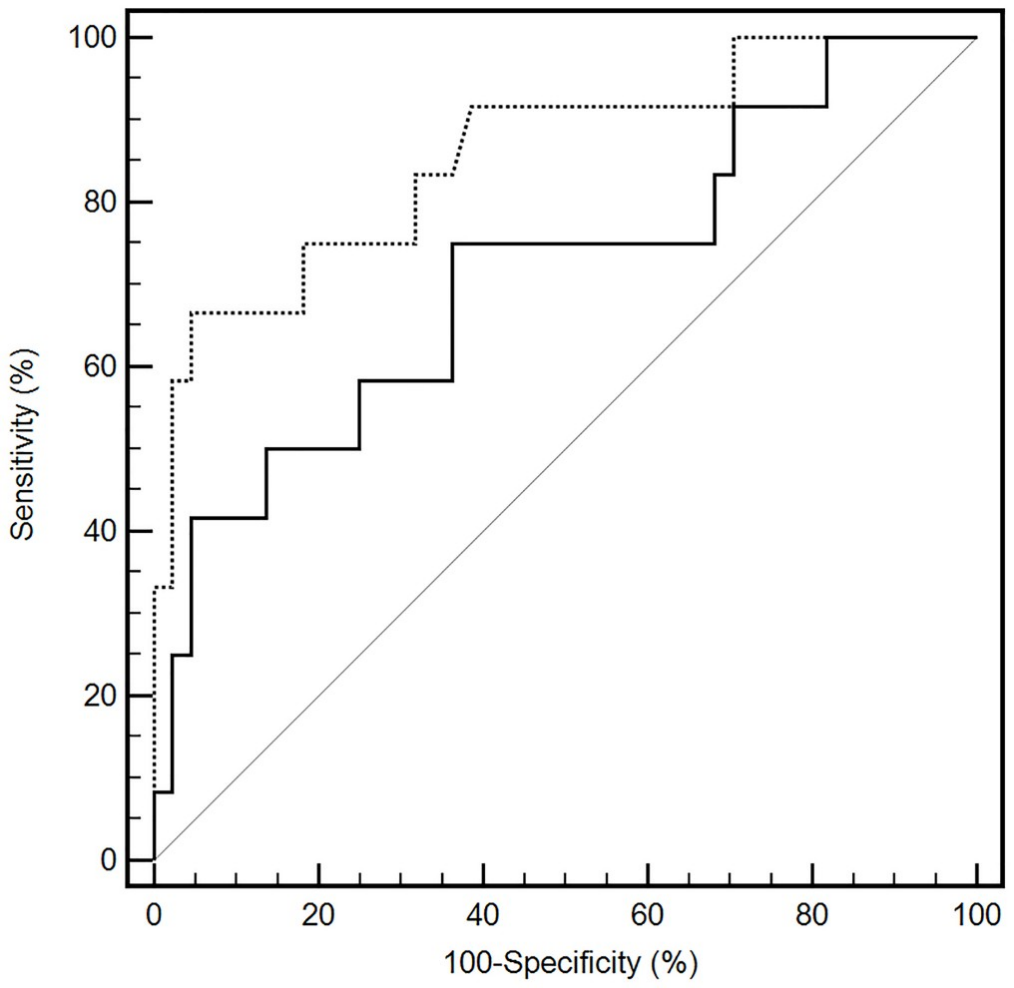
Receiver operating characteristic curves of the HOMA-Adiponectin and HOMA-IR for detecting insulin resistance. The dashed line represents the HOMA-IR (homeostasis model assessment of insulin resistance), and solid line represents the HOMA-Adiponectin (homeostasis model assessment of adiponectin). The solid grey line is the reference and it represents an AUC of 0.5.

The ROC curves of HOMA-Adiponectin and HOMA-IR in the detection of insulin resistance were presented in Fig 2. Both HOMA-Adiponectin (AUC: 0.712; 95% confidence interval [95CI%]: 0.576–0.825; *P* = 0.02) and HOMA-IR (AUC: 0.859; 95CI%: 0.740–0.937; *P* < 0.0001) presented good discriminatory power for the detection of insulin resistance. The AUC of HOMA-IR was significantly higher compared with HOMA-Adiponectin (difference between areas: 0.147; 95%CI: 0.042 to 0.251; *z* statistic: 2.766; *P* = 0.005).

## Discussion

To our knowledge, this is the first study in adolescents to examine the accuracy of HOMA-Adiponectin in detecting insulin resistance based on hyperglycaemic clamp technique. The main findings of our study are: 1) HOMA-Adiponectin was inversely associated with clamp-derived insulin sensitivity, but the variance of the insulin sensitivity explained by it was not superior to HOMA-IR; and 2) the HOMA-Adiponectin could discriminate patients with and without insulin resistance, however, without a better discriminatory accuracy than HOMA-IR.

HOMA-IR [1] has been validated as a surrogate measure of insulin resistance in children and adolescents and is widely used in epidemiological studies in pediatric populations [3, 7, 8, 23]. However, it assumes that hepatic and peripheral insulin resistance are equal. The inclusion of total serum adiponectin level in the denominator of the HOMA-IR may add an indirect measurement of adiposopathy [6] and, consequently, indirect information of peripheral insulin resistance, giving robustness to its pathophysiological basis.

Adiponectin is a protein synthesized mainly in adipose tissue and secreted into the serum [24]. One of the hypotheses for insulin-sensitizing effect by adiponectin is the increase in fatty-acid oxidation in muscle and liver via activation of adenosine monophosphate activated protein kinase (AMPK) and of peroxisome proliferator-activated receptor-alpha (PPAR-α), mediated by adiponectin receptors (AdipoR1 mainly expressed in the skeletal muscle and AdipoR2 in the liver) [25, 26]. In adolescents, the positive association between adiponectin and insulin sensitivity has been well documented [4, 5, 27, 28], similar result was observed in the present study (r = 0.338; *P* = 0.01).

HOMA-Adiponectin was validated as a surrogate measure of insulin resistance in Japanese adults [2], and consistent with our results, studies in adults have found an inverse correlation between the HOMA-Adiponectin and the clamp-derived insulin sensitivity [2, 6, 29]. However, contrary to what we observed, these studies reported a higher correlation coefficient for HOMA-Adiponectin when compared to HOMA-IR. In addition, in the multiple linear regression model, we observed that HOMA-Adiponectin was still associated with clamp-derived insulin sensitivity after adjustment for sex, Tanner stage and waist circumference-to-height ratio. In our data, however, the variance of the insulin sensitivity explained by the HOMA-Adiponectin was not superior to HOMA-IR. Contrary to what we observed, in a cross-sectional study of Japanese adults of both sexes, Matsuhisa et al. [2] observed that in the stepwise multivariate regression analysis the HOMA-Adiponectin was most significantly associated and independently with the clamp-derived insulin sensitivity (M-value calculated from euglycemic hyperinsulinemic clamp technique) than HOMA-IR. This disparity might be due to differences in populations studied (such as age and race/ethnicity), or methodological or in the statistical analyses.

Furthermore, the present study showed, for the first time, that HOMA-Adiponectin (AUC = 0.712) could discriminate patients with and without insulin resistance, however, without a better discriminatory accuracy than HOMA-IR (AUC = 0.859). In opposition with ours results, a Brazilian adult’s study, by our research group, showed a similar performance for HOMA-Adiponectin and HOMA-IR to identify insulin resistance [6]. The divergence in the results could be due to the group age, and to the criteria used for insulin resistance. In the present study, insulin resistance was defined based on cutoff of clamp-derived insulin sensitivity index (lower than the 10th percentile) derived from the normal-weight group.

Makni et al [30] in another cross-sectional study of obese children (mean age 13.7 ± 1.3 years), investigated the efficacy of HOMA-Adiponectin and HOMA-IR in assessing insulin resistance classified according to metabolic syndrome diagnosis. They reported an AUC of 0.71 (CI 95%: 0.62–0.79, *P* < 0.05) for the HOMA-Adiponectin and of 0.68 (CI 95%: 0.59–0.76, *P* < 0.01) for the HOMA-IR, and concluded that HOMA-Adiponectin is an adequate tool for determining insulin resistance among obese children with metabolic syndrome. However, the HOMA-Adiponectin was not compared with the glucose clamp technique, which is the reference method to assess insulin resistance.

Our results suggest that HOMA-Adiponectin could be used as a surrogate measure of insulin resistance in adolescents. However, we recommend the use of HOMA-IR in this age group since it presented better diagnostic accuracy than HOMA-Adiponectin in detecting insulin resistance. In addition, laboratory assays of plasma glucose and insulin are easier and cheaper to obtain than adiponectin measurement, which is not yet a routinely run test in public health service. Furthermore, previously, we have identified other more simple and practical methods, such as the waist circumference [8], sagittal abdominal diameter [8] and the hypertriglyceridemic waist phenotype (presence of a concomitant increase in plasma triglycerides and waist circumference) [7] that also can be used as surrogate measures of insulin resistance in this age group. The choice of the method will depend on the goals you are interested, research or clinical evaluation, the infrastructure available, the professional experience and the health service.

This study had some limitations. First, it is a cross-sectional study, so it does not allow causal inferences. Second, we used the hyperglycaemic clamp technique, not the gold standard, even because studies have reported a strong correlation between insulin sensitivity from this clamp technique and from the euglycaemic-hyperinsulinaemic clamp technique, the gold standard, in children and adolescents, with fewer side effects [15, 31, 32]. Third, sexual maturation was determined by self-assessment to increase the participation rate [10, 11], due to privacy concerns, cultural and emotional factors. Validation studies found reasonable agreement between self-assessed sexual maturation and physical examination performed by a physician in epidemiologic studies [10, 33], and suggest the self-assessed examination when the physician exam is not possible [11, 33]. In addition, Rasmussen et al [11] suggest that its use can be sufficiently accurate for a simple distinction between prepuberty and puberty for large epidemiologic studies. Fourth, we did not consider race/ethnicity because Brazilian people is one of the most admixture populations in the world.

In conclusion, our results showed that the HOMA-Adiponectin was independently associated with insulin resistance and exhibited a good discriminatory power for predicting it. However, it did not show superiority over HOMA-IR in the diagnostic accuracy.

## Supporting information

S1 File BRAMS dataset. In this part, we presented the dataset used for the statistical analyses. Participants were classified as pubertal (Tanner II-IV) and postpubertal (Tanner V) according to Tanner criteria [12]. The nutritional status was classified as normal weight (BMI-for-age at or above the 5th percentile and below the 85th percentile); overweight (BMI-for-age at or above the 85th percentile and below the 95th percentile); and obesity (BMI-for-age at or above the 95th percentile) according to the Centers for Disease Control and Prevention criteria (CDC/2000) [9].
(XLS)

S2 File Analysis codes. In this part, we presented the codes for each statistical test.
(DOC)
