# Supplementary material for: Homeostatic model assessment of adiponectin (HOMA-Adiponectin) as a surrogate measure of insulin resistance in adolescents: Comparison with the hyperglycaemic clamp and homeostatic model assessment of insulin resistance
Source: PLoS One. 2019 Mar 25;14(3):e0214081. doi: 10.1371/journal.pone.0214081 (PMC6433366; doi:10.1371/journal.pone.0214081)
Supplement: S2 File — In this part, we presented the codes for each statistical test. (DOC) [file pone.0214081.s002.doc]

S2 File - Analysis codes


Test of Normality - Shapiro-Wilk test
SORT CASES BY Nutritional_status.
SPLIT FILE LAYERED BY Nutritional_status.
EXAMINE VARIABLES=Age_years Weight_kg Height_cm BMI_kg_m2 Body_mass_index_for_age_percentile 
    Waist_circumference_cm Waist_to_height_ratio Lean_body_mass_kg Adiponectin_µg_mL 
    Plasma_glucose_mg_dL Plasma_insulin_mU_L HOMA_Adiponectin HOMA_IR
  /PLOT NPPLOT
  /STATISTICS DESCRIPTIVES
  /CINTERVAL 95
  /MISSING LISTWISE
  /NOTOTAL.


T-Test
SPLIT FILE OFF. 
T-TEST GROUPS=Nutritional_status(0 1) 
  /MISSING=ANALYSIS 
  /VARIABLES=Age_years Plasma_glucose_mg_dL Height_cm 
  /CRITERIA=CI(.95).
  

Custom Tables
* Custom Tables.
CTABLES
  /VLABELS VARIABLES=Weight_kg BMI_kg_m2 Body_mass_index_for_age_percentile Waist_circumference_cm 
    Waist_to_height_ratio Lean_body_mass_kg Adiponectin_µg_mL Plasma_insulin_mU_L HOMA_Adiponectin 
    HOMA_IR ISI_mg_kgLBM_1_min_1_per_mU_L_x100 Nutritional_status 
    DISPLAY=LABEL
  /TABLE Weight_kg [MEDIAN, PTILE 25, PTILE 75] + BMI_kg_m2 [MEDIAN, PTILE 25, PTILE 75] + 
    Body_mass_index_for_age_percentile [MEDIAN, PTILE 25, PTILE 75] + Waist_circumference_cm [MEDIAN, 
    PTILE 25, PTILE 75] + Waist_to_height_ratio [MEDIAN, PTILE 25, PTILE 75] + Lean_body_mass_kg 
    [MEDIAN, PTILE 25, PTILE 75] + Adiponectin_µg_mL [MEDIAN, PTILE 25, PTILE 75] + Plasma_insulin_mU_L 
    [MEDIAN, PTILE 25, PTILE 75] + HOMA_Adiponectin [MEDIAN, PTILE 25, PTILE 75] + HOMA_IR [MEDIAN, 
    PTILE 25, PTILE 75] + ISI_mg_kgLBM_1_min_1_per_mU_L_x100 [MEDIAN, PTILE 25, PTILE 75] BY 
    Nutritional_status
  /CATEGORIES VARIABLES=Nutritional_status ORDER=A KEY=VALUE EMPTY=INCLUDE.


NPar Tests
Mann-Whitney Test
NPAR TESTS
  /M-W= Weight_kg BMI_kg_m2 Body_mass_index_for_age_percentile Waist_circumference_cm 
    Waist_to_height_ratio Lean_body_mass_kg Adiponectin_µg_mL Plasma_insulin_mU_L HOMA_Adiponectin 
    HOMA_IR ISI_mg_kgLBM_1_min_1_per_mU_L_x100 BY Nutritional_status(0 1)
  /MISSING ANALYSIS.


Frequencies
SORT CASES BY Nutritional_status.
SPLIT FILE LAYERED BY Nutritional_status.
FREQUENCIES VARIABLES=Sex Tanner_stage
  /ORDER=ANALYSIS.


Fisher's Exact Test
SPLIT FILE OFF.
CROSSTABS
  /TABLES=Sex BY Nutritional_status
  /FORMAT=AVALUE TABLES
  /STATISTICS=CHISQ RISK 
  /CELLS=COUNT
  /COUNT ROUND CELL.


CROSSTABS
  /TABLES=Tanner_stage BY Nutritional_status
  /FORMAT=AVALUE TABLES
  /STATISTICS=CHISQ RISK 
  /CELLS=COUNT
  /COUNT ROUND CELL.


Correlations
CORRELATIONS
  /VARIABLES=Log10_ISI_mg_kgLBM_1_min_1_per_mU_L_x100 Log10_HOMA_adiponectin
  /PRINT=TWOTAIL NOSIG
  /MISSING=PAIRWISE.
  

CORRELATIONS
  /VARIABLES=Log10_ISI_mg_kgLBM_1_min_1_per_mU_L_x100 Log10_HOMA_IR
  /PRINT=TWOTAIL NOSIG
  /MISSING=PAIRWISE.

  


Regression
REGRESSION
  /DESCRIPTIVES MEAN STDDEV CORR SIG N
  /MISSING LISTWISE
  /STATISTICS COEFF OUTS CI(95) R ANOVA COLLIN TOL CHANGE
  /CRITERIA=PIN(.05) POUT(.10)
  /NOORIGIN 
  /DEPENDENT Log10_ISI_mg_kgLBM_1_min_1_per_mU_L_x100
  /METHOD=ENTER Log10_HOMA_adiponectin Sex Tanner_stage
  /SCATTERPLOT=(*ZRESID ,*ZPRED)
  /RESIDUALS HISTOGRAM(ZRESID) NORMPROB(ZRESID)
  /CASEWISE PLOT(ZRESID) OUTLIERS(2)
  /SAVE PRED ZPRED COOK RESID ZRESID.


Test of Normality of Residuals - Shapiro-Wilk test
EXAMINE VARIABLES=ZRE_1
  /PLOT HISTOGRAM NPPLOT
  /STATISTICS DESCRIPTIVES
  /CINTERVAL 95
  /MISSING LISTWISE
  /NOTOTAL.


Regression
REGRESSION
  /DESCRIPTIVES MEAN STDDEV CORR SIG N
  /MISSING LISTWISE
  /STATISTICS COEFF OUTS CI(95) R ANOVA COLLIN TOL CHANGE
  /CRITERIA=PIN(.05) POUT(.10)
  /NOORIGIN 
  /DEPENDENT Log10_ISI_mg_kgLBM_1_min_1_per_mU_L_x100
  /METHOD=ENTER Log10_HOMA_adiponectin Sex Tanner_stage Waist_to_height_ratio
  /SCATTERPLOT=(*ZRESID ,*ZPRED)
  /RESIDUALS HISTOGRAM(ZRESID) NORMPROB(ZRESID)
  /CASEWISE PLOT(ZRESID) OUTLIERS(2)
  /SAVE PRED ZPRED COOK RESID ZRESID.


Test of Normality of Residuals - Shapiro-Wilk test
EXAMINE VARIABLES=ZRE_2
  /PLOT HISTOGRAM NPPLOT
  /STATISTICS DESCRIPTIVES
  /CINTERVAL 95
  /MISSING LISTWISE
  /NOTOTAL.


Regression
REGRESSION
  /DESCRIPTIVES MEAN STDDEV CORR SIG N
  /MISSING LISTWISE
  /STATISTICS COEFF OUTS CI(95) R ANOVA COLLIN TOL CHANGE
  /CRITERIA=PIN(.05) POUT(.10)
  /NOORIGIN 
  /DEPENDENT Log10_ISI_mg_kgLBM_1_min_1_per_mU_L_x100
  /METHOD=ENTER Log10_HOMA_IR Sex Tanner_stage
  /SCATTERPLOT=(*ZRESID ,*ZPRED)
  /RESIDUALS HISTOGRAM(ZRESID) NORMPROB(ZRESID)
  /CASEWISE PLOT(ZRESID) OUTLIERS(2)
  /SAVE PRED ZPRED COOK RESID ZRESID.


Test of Normality of Residuals - Shapiro-Wilk test
EXAMINE VARIABLES=ZRE_3
  /PLOT HISTOGRAM NPPLOT
  /STATISTICS DESCRIPTIVES
  /CINTERVAL 95
  /MISSING LISTWISE
  /NOTOTAL.


Regression
REGRESSION
  /DESCRIPTIVES MEAN STDDEV CORR SIG N
  /MISSING LISTWISE
  /STATISTICS COEFF OUTS CI(95) R ANOVA COLLIN TOL CHANGE
  /CRITERIA=PIN(.05) POUT(.10)
  /NOORIGIN 
  /DEPENDENT Log10_ISI_mg_kgLBM_1_min_1_per_mU_L_x100
  /METHOD=ENTER Log10_HOMA_IR Sex Tanner_stage Waist_to_height_ratio
  /SCATTERPLOT=(*ZRESID ,*ZPRED)
  /RESIDUALS HISTOGRAM(ZRESID) NORMPROB(ZRESID)
  /CASEWISE PLOT(ZRESID) OUTLIERS(2)
  /SAVE PRED ZPRED COOK RESID ZRESID.


Test of Normality of Residuals - Shapiro-Wilk test
EXAMINE VARIABLES=ZRE_4
  /PLOT HISTOGRAM NPPLOT
  /STATISTICS DESCRIPTIVES
  /CINTERVAL 95
  /MISSING LISTWISE
  /NOTOTAL.


Receiver operating characteristic (ROC) analysis
Statistics
ROC curves
ROC curves analysis
Variable HOMA_Adiponectin
Classification variable Insulin_resistance
Methodology DeLong et al.
Binominal exact Confidence Interval for the AUC


Statistics
ROC curves
ROC curves analysis
Variable HOMA_IR
Classification variable Insulin_resistance
Methodology DeLong et al.
Binominal exact Confidence Interval for the AUC


Comparison between areas under the ROC curve (AUC)
Statistics
ROC curves
Comparison of ROC curves
Variables HOMA_Adiponectin HOMA_IR
Classification variable Insulin_resistance
Methodology DeLong et al.
Binominal exact Confidence Interval for the AUC
